# Accurate quantitative myocardial perfusion using single cycle T1 mapping

**DOI:** 10.1186/1532-429X-15-S1-W5

**Published:** 2013-01-30

**Authors:** D Chen, B Sharif, R Dharmakumar, D Li

**Affiliations:** 1 BIRI, Cedars Sinai Hospital, Los Angeles, CA, USA

## Background

Quantitative myocardial perfusion MRI depends on accurate determination of the arterial input function (AIF). Unfortunately, typical doses of contrast agent (CA) cause saturation in the AIF signal intensity at peak CA concentrations. Methods using dual boluses [1] or dual sequences [2] have been proposed to correct for this effect; however, they require additional data acquisition which complicates the imaging procedure. CA concentrations can be directly found by calculating blood and myocardial T1 values [3,4]. This method was performed in vivo and compared to the dual sequence method [2].

## Methods

Radial AIF correction was performed using the method described in [4]. T1 relaxation following an SR magnetization preparation and FLASH acquisition can be modeled [3]. By acquiring multiple images after a single SR and using the acquisition parameters, pixelwise T1 can be found by solving a simple non-linear data fitting exercise. Gd concentration is found using the known relaxivity of Gd CA [6].

Ten healthy volunteers underwent perfusion MRI studies on a Siemens 3T Verio system with IRB approval and written consent. Two first pass perfusion scans were performed at rest using a radial acquisition for T1 mapping and a dual TI Cartesian acquisition. The Cartesian scan had a resolution of 3×2×8 mm3 and TI = 120 ms. The short TI scan has TI = 28 ms. The multi-shot radial scan had a resolution of 1.7×1.7×8 mm3; 3 images per SR with TI = 50, 94, and 138 ms respectively. Reconstruction of radial data was performed using a compressed sensing method with a composite reference constraint [3].

## Results

**Figure 1:**
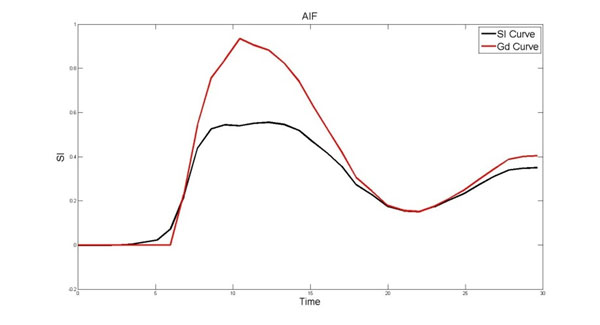
AIF Curves produced from the radial scan. The black curve shows the AIF derived from raw SI. The red curve shows the AIF taken from Gd concentrations produced from the described method. The peak signal amplitude is recovered by finding the Gd concentration.

Results: Raw Cartesian and radial perfusion signal intensity curves showed higher than expected rest MBF. Using the proposed method, the Gd concentration of the AIF was found. Fig. 1 shows signal curves comparing uncorrected radial and corrected. Using the proposed T1 mapping technique allows for correction of the radial AIF (‘SI Curve’). The MBF using the corrected AIF compared favorably with MBFs found using the AIF from the dual sequence, as presented in Table 1.

**Table 1:** Comparisons of MBF (ml/min/g) measurements using Cartesian and radial techniques. There is no significant difference between radially acquired and Cartesian MBFs.

	Cartesian	Radial	P-Value
Uncorrected	1.96 ± 0.45	1.88 ± 0.41	P<0.33
Corrected	1.34 ± 0.41	1.35 ± 0.27	P<0.23

## Conclusions

Myocardial perfusion measurement using radial T1-mapping eliminates the need to acquire two datasets to produce an unsaturated AIF with the dual sequence approach, making it more practical for clinical use. The major problem with the dual sequence method is it produces AIF of different signal intensity scale due to different T1 weighting. The AIF needs to be scaled properly with respect to myocardial tissue function for accurate MBF measurements. The proposed method allows the myocardial tissue signal and AIF to be extracted using the same set of the images, thus eliminating a potential source of error.

## Funding

NIH Grant T32 EB51705

NIH Grant RO1 EB002623

AHA Postdoctoral Fellowship Award 1POST7390063
